# Metabolic Fingerprint of Turner Syndrome

**DOI:** 10.3390/jcm9030664

**Published:** 2020-03-02

**Authors:** Jolanta Bugajska, Joanna Berska, Małgorzata Wójcik, Jerzy B. Starzyk, Krystyna Sztefko

**Affiliations:** 1 Department of Clinical Biochemistry, Institute of Pediatrics, Jagiellonian University Medical College, Krakow, Wielicka St. 265, 30-663 Krakow, Poland; joanna.berska@uj.edu.pl (J.B.); misztefk@cyf-kr.edu.pl (K.S.); 2 Department of Paediatric and Adolescent Endocrinology, Institute of Paediatrics, Jagiellonian University, Medical College, Krakow, Wielicka St. 265, 30-663 Krakow, Poland; malgorzata.wojcik@uj.edu.pl (M.W.); jerzystarzyk@cm-uj.krakow.pl (J.B.S.)

**Keywords:** Turner syndrome, amino acids, branched-chain amino acids, obesity, metabolomics

## Abstract

Girls with Turner syndrome (TS) are at increased risk of developing insulin resistance and coronary artery disease as a result of hypertension and obesity frequently seen in these patients. On the other hand, it is known that obesity is associated with increased serum levels of branched-chain amino acids (BCAAs: valine; leucine and isoleucine) and aromatic amino acids. The aim of the study is to compare the metabolic fingerprint of girls with TS to the metabolic fingerprint of girls with obesity. Metabolic fingerprinting using an untargeted metabolomic approach was examined in plasma from 46 girls with TS (study group) and 22 age-matched girls with obesity (control group). The mean values of BCAAs, methionine, phenylalanine, lysine, tryptophan, histidine, tyrosine, alanine and ornithine were significantly lower in the study group than in the control (*p* from 0.0025 to <0.000001). Strong significant correlation between BCAAs, phenylalanine, arginine, tyrosine, glutamic acid, citrulline and alanine, and body mass index expressed as standard deviation score BMI-SDS in the patients with obesity (*p* from 0.049 to 0.0005) was found. In contrast; there was no correlation between these amino acids and BMI-SDS in the girls with TS. It is suggested that obesity in patients with TS is not associated with altered amino acids metabolism.

## 1. Introduction

Turner syndrome (TS) is a congenital disease caused by absence or structural abnormalities of sex chromosomes, resulting in short stature and gonadal dysgenesis. Body composition is altered in women with TS. Adult women with Turner syndrome have decreased muscle mass and increased total fat mass, including visceral fat mass [1]. Women with Turner syndrome are at increased risk of developing insulin resistance syndrome and coronary artery disease as a result of hypertension and obesity, frequently seen in these patients [1,2,3,4]. Growth hormone (GH) treatment in girls with TS has a beneficial effect for body composition and lipid profile, but it may affect glucose metabolism and increase insulin resistance [5,6]. It is known that most patients who develop type 2 diabetes during GH treatment have preexisting risk factors for impairment of glucose homeostasis [7].

The comprehensive metabolite profile of any biological sample is provided by metabolomics. The metabolic fingerprint in patients with obesity is associated with changes in serum amino acid profile. Results of studies have shown increased serum levels of branched chain amino acids (BCAAs: valine, leucine and isoleucine), aromatic amino acids (phenylalanine, tyrosine and tryptophan) and some others like lysine, cysteine and glutamate in patients with obesity [8,9]. These amino acids are assumed as predictors of the future development of diabetes. It is noteworthy that a single, fasting measurement of branched-chain and aromatic amino acids may provide firmer information than standard risk factors (such as BMI, dietary patterns and fasting glucose) [10]. It is suggested that, based on increased level of circulating branched-chain amino acids in overweight individuals, hepatic insulin resistance and organ-specific fat storage might be predicted [11].

The aim of the study is to compare metabolic fingerprint of girls with TS to metabolic fingerprint of girls with obesity.

## 2. Methods

Forty-six girls with Turner syndrome 21 with X chromosome monosomy (45,X), 8 with abnormal X chromosome and 17 with mosaicism, were selected for the study group (mean age: 12.4 ± 4.2 years). 36 of them were receiving growth hormone therapy. In addition, 14 out of 46 girls with TS were on estrogen replacement therapy. Spontaneous puberty was observed in 5/46 of the patients. The study group consisted of 32 patients without obesity and 14 patients with obesity. The control group consisted of 22 girls with obesity (mean age: 14.0 ± 2.9 years). Body mass index (BMI) was expressed as SDS (Standard Deviation Score) according to the Polish National Standards [12]. There was no significant difference concerning mean age in both groups (*p* < 0.11) but significant difference was shown for BMI (*p* < 0.000001) (Table 1). The mean value of BMI SDS in non-obese patients with TS was 0.06 ± 0.70 and in obese patients with TS was 3.11 ± 1.49 (*p* < 0.0002). There was no significant difference between mean value of BMI SDS between obese patients with TS and patients with obesity (*p* = 0.12).

From each patient fasting venous blood sample was drawn on lithium heparin and into tubes containing separating gel. The blood was centrifuged for 10 min at 1200× *g*, and plasma and serum were kept at −70 °C until analysis.

The determination of free amino acids (AA) plasma profile was performed using the fully validated, highly selective liquid chromatography-tandem mass spectrometry method (LC-MS/MS) with Jasem quantitative amino acids analysis kit. Samples were analyzed by a high-performance liquid chromatograph 1260 Infinity II (Agilent Technologies, Waldbronn, Germany) interfaced to a triple-quadrupole mass spectrometer 6460 QTRAP (Agilent Technologies, Singapore. The MassHunter program (Agilent Technologies, Waldbronn, Germany) was used to collect and compile data. The essential amino acids: valine, isoleucine, leucine, threonine, methionine, phenylalanine, lysine, tryptophan, histidine, and the nonessential amino acids: arginine, tyrosine, aspartic acid, glutamic acid, serine, asparagine, glycine, glutamine, taurine, citrulline, alanine, proline and ornithine were determined. 

Serum free fatty acids (FFAs) were determined by a colorimetric method using a commercial kit (Randox Laboratories Ltd., CrumLin, United Kingdom). Insulin concentrations were measured using an ADVIA Centaur® XP analyzer according to the manufacturer’s instructions. Glucose concentrations were measured by dry chemistry analyzer (Vitros 4600, Ortho Clinical Diagnostics Inc., Rochester, NY, USA). HOMA-IR was calculated using the formula: fasting insulin level (µIU/mL) × fasting glucose level (mmol/L)/22.5. Insulin resistance definition was based on a HOMA-IR threshold set for adolescents ≥ 3.16 [13].

The study was approved by the Jagiellonian University Bioethics Committee (Protocol No. 122.6120.35.2016).

Written consent was obtained from all parents before their children were included in the study. All methods performed in the study were conducted following all ethical and legal regulations.

### Statistical Analysis

Descriptive statistics (mean values, SD medians, quartiles Q1–Q3) were used in the statistical assessment of the results. Statistica software version 10 (StatSoft) and Microsoft Office Excel 2003 were used to perform statistical analysis. To evaluate the distribution of continuous variables in terms of compliance with the normal distribution, the Shapiro-Wilk test was employed. Students’ *t*-test was applied to compare the mean concentrations of amino acids between the study group and the control group for normally distributed continuous variable; in case of non-normal distribution, the Mann-Whitney U test was used. Pearson’s correlation was used to examine relationships between BMI-SDS and amino acids. A *p* value less than 0.05 was considered statistically significant.

## 3. Results

No significant differences in mean values of glucose, insulin and FFAs levels, as well as HOMA-IR, between TS patients on GH therapy and TS patients without GH therapy were noticed. There was also no significant difference in mean values of fasting glucose level between patients with TS and patients with obesity, but the values of insulin level and HOMA-IR were significantly lower in patients with TS. In contrast, the mean value of FFAs level was significantly higher in patients with TS than in patients with obesity (Table 2). The mean values of valine, isoleucine, leucine (branched-chain amino acids), methionine, phenylalanine, lysine, tryptophan, histidine, tyrosine, alanine and ornithine were significantly lower in the study group than in the control (*p* from 0.0025 to <0.000001), and the mean value of aspartic acid was significantly higher in patients with TS than in patients with obesity (*p* < 0.005) (Table 3).

**Figure 1:**
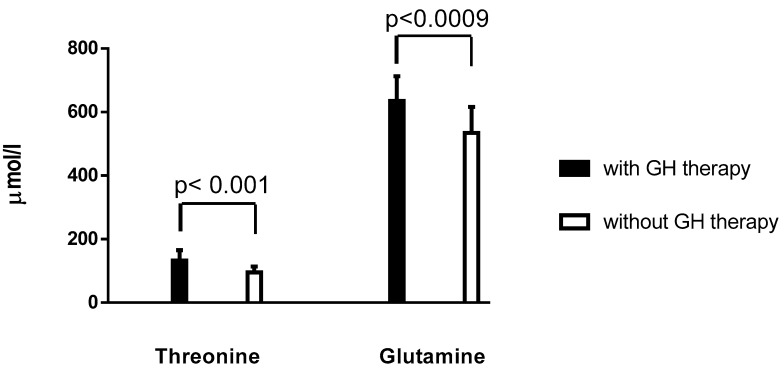
Fasting plasma mean concentrations of threonine and glutamine (±SD) between Turner syndrome (TS) patients with GH therapy and TS patients without GH therapy.

There was significant difference in the mean values of threonine and glutamine concentrations between TS patients with GH therapy and TS patients without GH therapy (*p* = 0.001 and *p* = 0.0009, respectively) (Figure 1). Similarly, mean values of threonine and glutamine were higher in non-obese TS patients with GH therapy than in non-obese TS patients without GH therapy (*p* = 0.002 and *p* = 0.007, respectively) and no differences in the mean values of other amino acid concentrations between these groups were noticed.

Amino acids profile has also been compared in girls with TS between three groups: in girls who were on estrogen replacement therapy, in girls without estrogen replacement therapy and in girls with spontaneous puberty. The mean values of threonine and alanine were higher in girls with TS on estrogen replacement therapy than in girls with TS without estrogen replacement therapy (threonine: 148.9 ± 32.3 µmol/L; 108.7 ± 30.2 µmol/L; *p* = 0.033; alanine 407.0 ± 84.8 µmol/L; 300.9 ± 78.2 µmol/L *p* = 0.0039; respectively). No differences in the mean values of other amino acid concentrations were noticed.

**Figure 2:**
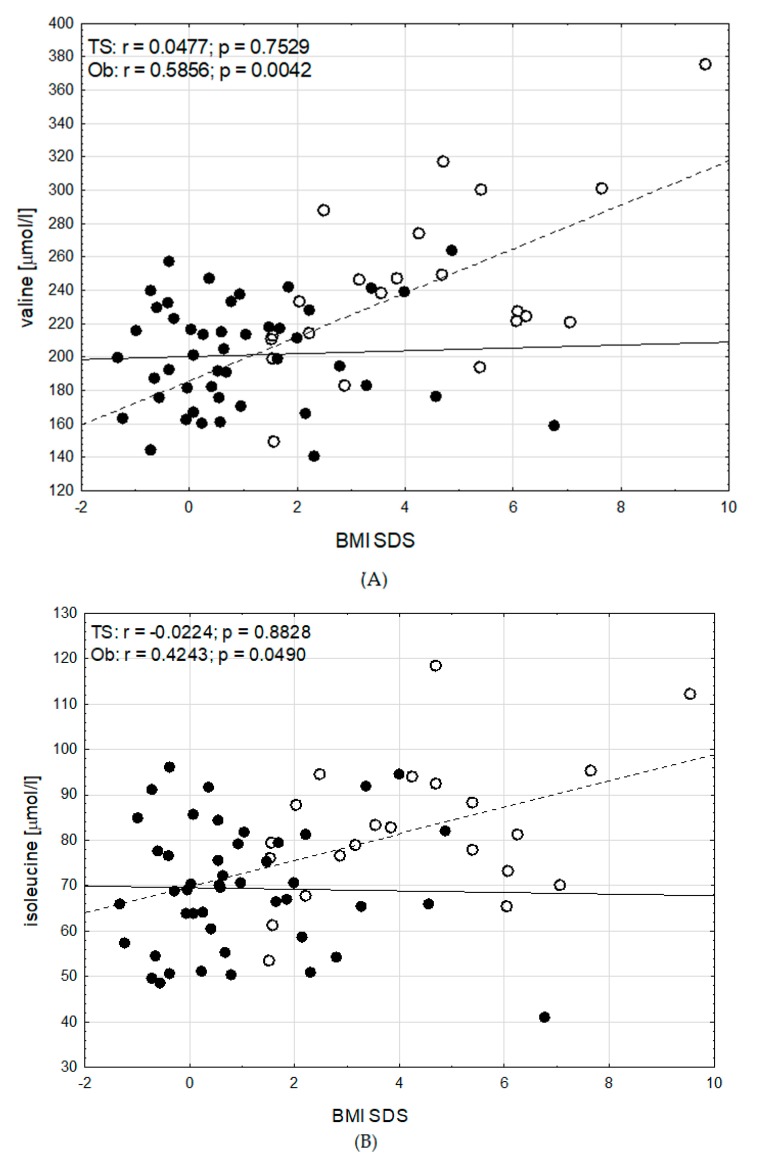

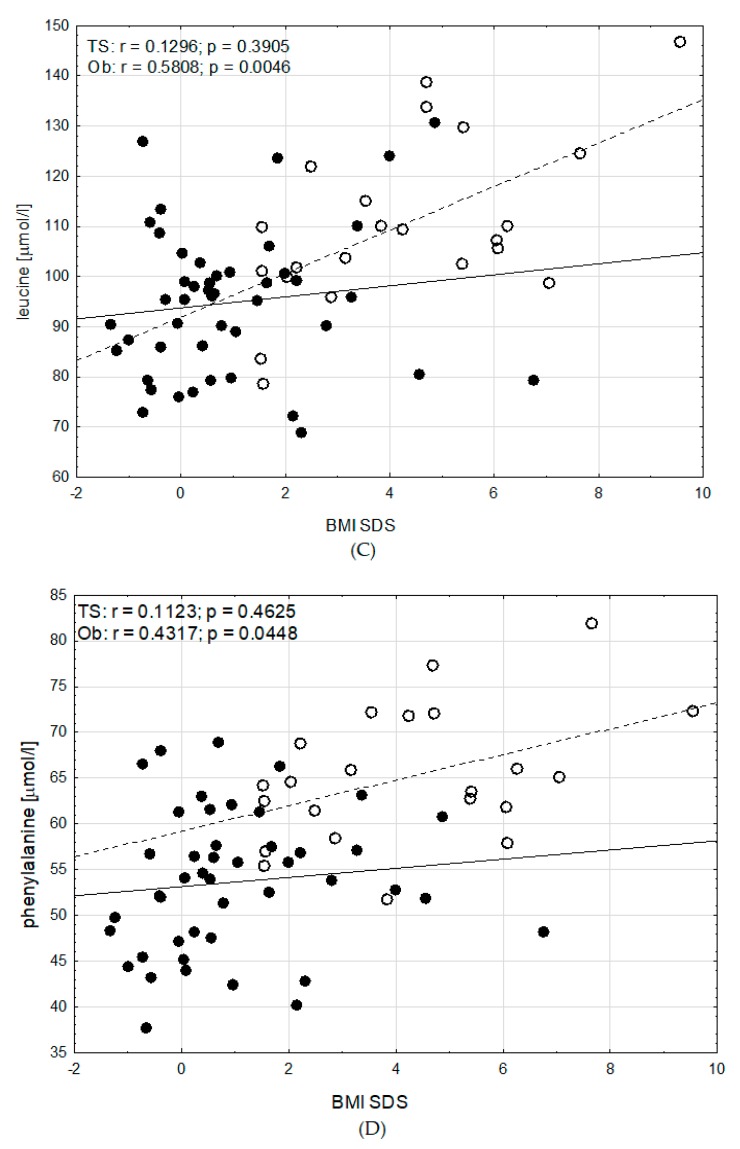
Correlations between (**A**) valine, (**B**) isoleucine, (**C**) leucine and (**D**) phenylalanine concentrations, and BMI SDS in girls with Turner syndrome (TS) (• and ━) and girls with obesity (Ob) (○ and - - -).

**Figure 3:**
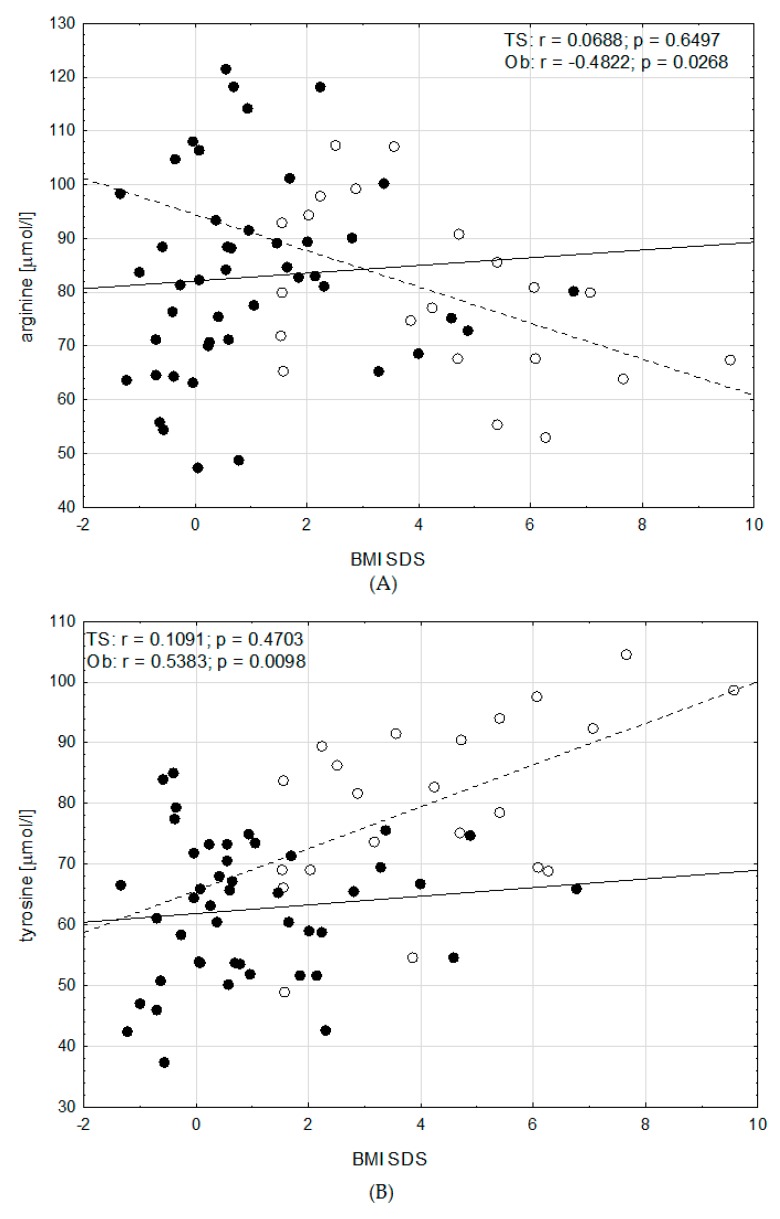

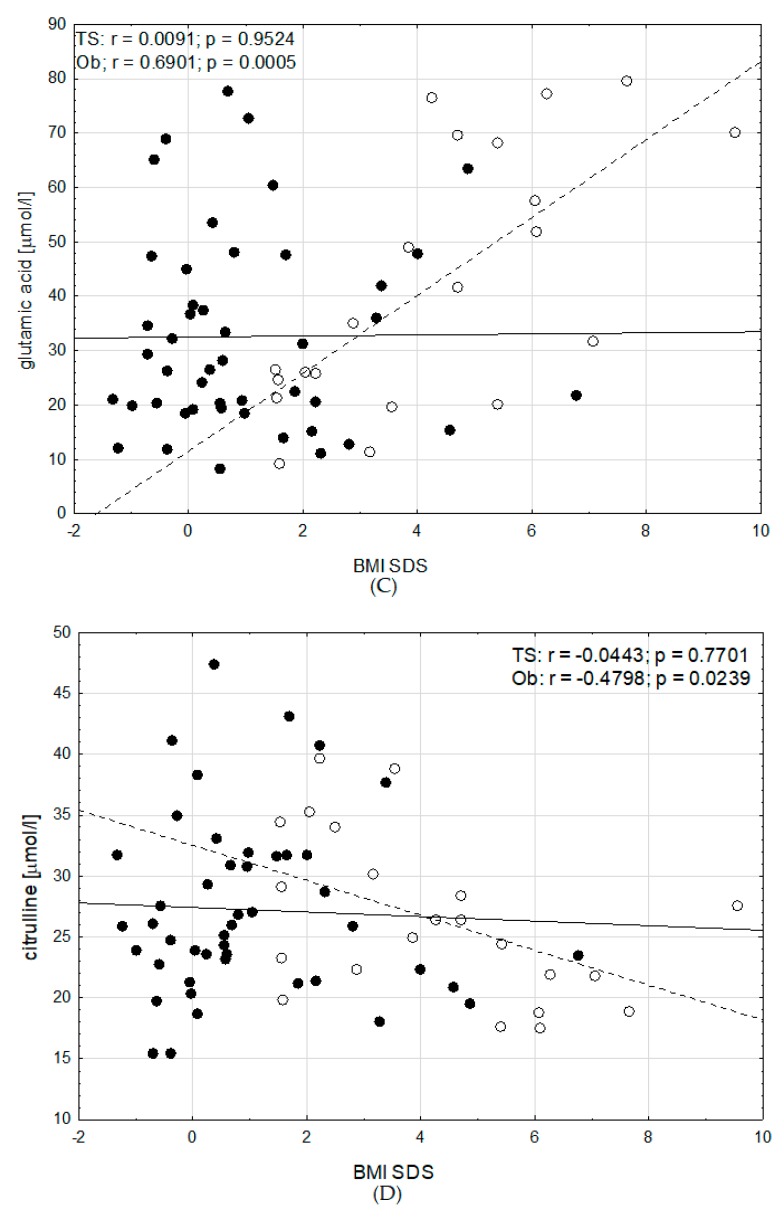

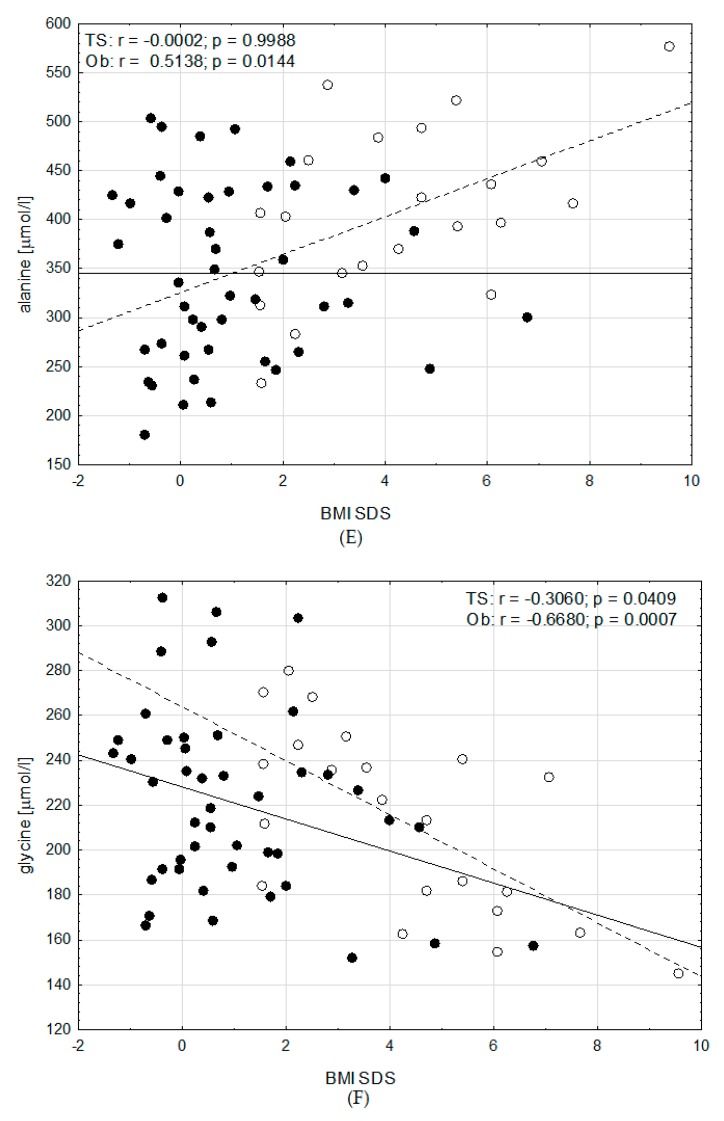
Correlations between non-essential amino acid concentrations: (**A**) arginine, (**B**) tyrosine, (**C**) glutamic acid, (**D**) citrulline, (**E**) alanine, (**F**) glycine, and BMI SDS in girls with TS (• and ━) and girls with Ob (○ and - - -).

We analysed correlations between BMI SDS and amino acids. There were the same relations between amino acids and BMI SDS in girls with and without GH therapy, therefore we checked correlations between BMI SDS and amino acids in all patients with TS, regardless of the GH hormone therapy. We found a strong significant correlation between valine, isoleucine, leucine, phenylalanine, arginine, tyrosine, glutamic acid, citrulline and alanine concentrations, and BMI SDS in the patients with obesity (r = 0.59, *p* < 0.005; r = 0.42, *p* < 0.05; r = 0.58, *p* < 0.005; r = 0.43, *p* < 0.05; r = 0.48, *p* < 0.03; r = 0.54, *p* < 0.01; r = 0.69, *p* < 0.0006; r = −0.48, *p* < 0.03; r = 0.51, *p* < 0.02; respectively). In contrast, there was no correlation between these amino acids and BMI SDS in girls with TS (Figure 2 and Figure 3). Glycine correlated significantly and negatively with BMI SDS, both in girls with obesity (r = −0.67, *p* < 0.0008) and in girls with TS (r = −0.31, *p* < 0.05) (Figure 3).

## 4. Discussion

Turner syndrome is a rare disease. Some patients with TS are receiving growth hormone therapy or estrogen/progesterone replacement therapy and some of them are obese. It is very difficult to perform an investigation in girls with TS free of such standard care and treatment. All patients with TS who were involved in our study were under standard treatment that may make our observations more important for clinical practice. Additional information about biochemical markers may be helpful in prevention of obesity in patients with TS.

Replacement therapy with a low-dose GH in GH-deficient adult subjects is associated with a sustained deterioration of glucose metabolism as a consequence of the lipolytic effect of GH. This could reflect a switch from glucose metabolism to lipids metabolism [14]. Although, the GH doses used in TS are significantly higher than in replacement therapy, our results showed no differences in mean values of FFAs between TS patients with GH therapy and TS patients without GH therapy. These observations agree with the results obtained by Bramnert et al. [14], who did not observe any significant changes in FFAs concentrations during GH treatment in adult women and men, compared with state before treatment or placebo. Sas et al. [15] showed that long term GH treatment in girls with TS has no adverse effect on glucose metabolism, but induced higher levels of insulin, indicating relative insulin resistance. We showed no significant difference in the mean glucose and insulin levels between TS patients with GH therapy and TS patients without GH therapy, but mean level of insulin was higher in TS patients with GH therapy than in TS patients without GH therapy. The lack of statistical significance may be due to the small size of the group without GH therapy.

Results of different studies regarding the impact of GH replacement therapy on changes in plasma amino acids concentration are conflicting. Fernholm et al. [16] did not find significant changes in AA levels in plasma in either males or females after 12 months of GH replacement therapy. Lundeberg et al. [17] in healthy male volunteers showed a higher concentration of glutamine, alanine and lower valine, leucine and histidine in plasma in the GH-treated group as compared with the initial values. In our study, we found higher concentrations of mean value of glutamine and threonine in TS patients with GH therapy than in TS patients without GH therapy. Glutamine participates in many key metabolic processes, such as protein synthesis, gluconeogenesis, inter-organ nitrogen transfer, nucleic acid biosynthesis, the immune response and regulation of cellular redox state. Threonine is particularly important for mucin synthesis and maintenance of gut barrier integrity [18].

There are no studies of metabolomics profiles in girls with TS who were on estrogen replacement therapy. Our results may only be compared to metabolomics profiles in postmenopausal women on replacement therapy. Our study has shown higher level of threonine in girls with TS who were on estrogen replacement therapy. Similar results were obtained by Stevens et al. [19] and Zang et al. [20] in postmenopausal women on estrogen replacement therapy. Further investigations are necessary to evaluate amino acids profile in girls with TS treated with GH and estrogen replacement therapy.

The present metabolomics analysis of patients with obesity revealed changes in the amino acids profile. Our results showed differences concerning the mean concentration of fasting branched-chain amino acids, methionine, phenylalanine, lysine, tryptophan, histidine, tyrosine, aspartic acid, alanine and ornithine between patients with obesity and patients with Turner syndrome. These amino acids are linked to obesity, insulin resistance and type 2 diabetes [8,9,10,11,21,22,23,24,25,26]. Leucine regulates a large array of cellular processes in pancreatic β-cells, including growth, proliferation and insulin secretion, which ultimately influence overall glucose homeostasis [21,22]. Glycine and serine were lower, whereas alanine, aspartate, cysteine, ornithine, phenylalanine, proline and tyrosine were higher in the young adults who were overweight or obese than in those with normal weight [23]. Methionine is capable of directly regulating gluconeogenesis. The transamination pathway of methionine metabolism was shown to control hepatic gluconeogenesis through the general control non-repressed protein 5 (GCN5) activity [24]. An animal study showed that methionine restriction protects from developing obesity, insulin resistance and type 2 diabetes [25,26]. BCAAs may make an independent contribution to development of insulin resistance and diabetes [27]. There are sparse data on BCAAs in patients with TS. Only Caprio et al. [28] demonstrated no significant differences for BCAAs between patients with Turner syndrome and healthy control. In the present study, these BCAAs concentrations were higher in patients with obesity than in patients with TS, which may be related to worse metabolic health and future insulin resistance or type 2 diabetes mellitus.

No data in the literature showed correlation between AA and BMI in patients with TS. Only in a few papers the correlation between AA and BMI was analysed. Strong positive correlation between aspartic acid, phenylalanine and BMI and negative correlation between glycine, serine, asparagine and BMI in young Mexican women has been shown [23]. Takashina et al. showed that plasma glycine and citrulline levels negatively correlated with BMI [9]. An animal study showed, that glycine reduces intra-abdominal fat accumulation through the increasing fatty acids oxidation in adipose tissue [29]. It has been suggested, that glycine and taurine supplementation may be an effective strategy for the prevention of high-fat diet induced obesity [30]. We observed the strong significant correlation between valine, isoleucine, leucine, phenylalanine, arginine, tyrosine, glutamic acid, citrulline, alanine and BMI SDS in patients with obesity, but there was no correlation between these amino acids and BMI SDS in girls with TS. Additionally, we found negative correlation between glycine and BMI SDS, both in girls with obesity and in girls with TS. For the girls with TS and obesity the decreasing levels of glycine with increasing levels of BMI SDS was noted. It can be speculated that glycine supplementation may be helpful for reducing obesity.

The limitation of the study is the small number of girls with TS without GH therapy available for the analysis. However, we do not think that it would influence the conclusion, because the differences in the metabolic fingerprint between the girls with TS and the girls with obesity are significant.

## 5. Conclusions

Due to differences in the metabolic fingerprint between girls with TS and girls with obesity, it is suggested, that obesity in patients with TS is not associated with altered amino acids metabolism.

## Figures and Tables

**Table 1:** Clinical characteristics.

Variables	Turner Syndrome	Children with Obesity	*p*
	Mean ± SD or Median (Interquartile Range)		
Age [years]	12.4 ± 4.2	14.0 ± 2.9	0.11
Body height [cm]	140.1 (123.3–149.6)	165.0 (152.0–171.3)	0.000001
Body mass [kg]	39.4 ± 15.7	80.1 ± 16.3	<0.000001
Body mass index [SDS]	0.56 (−0.28–1.85)	4.05 (2.23–6.06)	<0.000001

**Table 2:** Fasting serum concentrations of glucose, insulin, FFAs levels and HOMA-IR in the control group and in the study group.

Variables	Turner Syndrome		*p*	Turner Syndrome	Children with Obesity	*p*
	with Growth Hormone (GH) Therapy	without GH Therapy				
	n = 36	n = 10		n = 46	n = 22	
	Mean ± SD or Median (Interquartile Range)			Mean±SD or Median (Interquartile Range)		
Fasting glucose level [mmol/L]	4.69 ± 0.57	4.60 ± 0.32	0.63	4.67 ± 0.52	4.43 ± 0.39	0.06
Fasting insulin level [µIU/mL]	9.75 (6.7–13.8)	7.05 (3.7–12.2)	0.15	9.3 (6.3–13.45)	17.3 (11.6–24.8)	0.0008
Fasting FFAs level [mmol/L]	1.20 ± 0.62	0.98 ± 0.45	0.33	1.16 ± 0.59	0.85 ± 0.59	<0.05
HOMA-IR	1.95 (1.34–3.26)	1.38 (0.77–2.66)	0.15	1.92 (1.21–3.26)	3.3 (2.27–4.44)	0.005

**Table 3:** Fasting plasma concentrations of amino acids in the control group and in the study group.

Variables	Turner Syndrome		*p*	Turner Syndrome	Children with Obesity	*p*
	without Obesity	with Obesity				
	n = 32	n = 14		n = 46	n = 22	
	Mean ± SD or Median (Interquartile Range)			Mean ± SD or Median (Interquartile Range)		
**Essential amino acids [µmol/L]**						
Valine	199.9 ± 29.4	204.2 ± 36.4	0.68	201.2 ± 31.3	241.9 ± 50.7	0.0001
Isoleucine	69.5 ± 13.3	69.2 ±15.4	0.94	69.4 ± 13.8	82.2 ± 15.3	0.001
Leucine	93.2 ± 12.1	98.5 ± 19.3	0.27	94.8 ± 14.7	110.3 ± 16.8	0.0002
Threonine	124.2 ± 34.3	124.2 ± 33.6	0.99	124.2 ± 33.7	137.9 ± 32.6	0.12
Methionine	20.3 ± 3.7	19.9 ± 2.7	0.70	20.1 ± 3.4	24.2 ± 2.7	0.000007
Phenylalanine	53.4 ± 8.1	54.2 ± 7.2	0.76	53.6 ± 7.7	65.2 ± 7.3	<0.000001
Lysine	174.7 ± 26.8	183.4 ± 29.5	0.33	177.3 ± 27.6	216.4± 29.0	0.000002
Tryptophan	54.8 ± 12.1	57.7 ± 8.3	0.42	55.7 ± 11.1	71.0 ± 10.0	0.000001
Histidine	75.2 ± 8.4	80.7 ± 9.6	0.06	76.8 ± 9.0	84.7 ± 9.9	0.0025
**Non-essential amino acids [µmol/L]**						
Arginine	81.8 ± 19.7	85.2 ± 14.1	0.56	82.8 ± 18.1	80.0 ± 15.9	0.54
Tyrosine	62.8 ± 12.1	62.0 ± 9.6	0.83	62.6 ± 11.3	80.3 ± 14.4	0.00001
Aspartic acid	7.94 ± 1.68	7.49 ± 2.71	0.50	7.80 ± 2,04	6.35 ± 1.42	0.005
Glutamic acid	28.8 (20.2–46.3)	22.2 (15.2–41.9)	0.36	27.4 (19.5–45.1)	35.1 (24.7–68.3)	0.10
Serine	112.6 ± 22.9	101.6 ± 14.9	0.11	109.3 ± 21.3	114,3± 21.8	0.36
Asparagine	40.9 ± 7.6	39.3 ± 7.4	0.52	40.4 ± 7.5	41.2± 8.8	0.71
Glycine	226.9 ± 39.3	208.0 ± 42.6	0.15	221.1 ± 40.8	212.8 ± 40.5	0.44
Glutamine	617.0 ± 95.8	604.2 ± 72.2	0.66	613.1± 88.6	593.4 ± 75.6	0.38
Taurine	52.6 ± 12.0	47.3 ± 9.2	0.14	51.0 ± 11.3	49.7 ± 6.6	0.65
Citrulline	27.1 ± 7.0	27.6± 8.2	0.83	27.2 ± 7.3	26.4 ± 6.7	0.67
Alanine	343.2 ± 93.4	349.0 ± 80.9	0.84	344.9 ±88.9	408.1 ± 85.2	0.0007
Proline	196.4 ± 66.1	179.6± 58.5	0.42	191.3 ± 63.7	193.8 ± 53.7	0.87
Ornithine	41.6± 9.2	42.4 ± 9.8	0.81	41.9 ± 9.3	51.3 ± 11.6	0.0006

## Data Availability

The datasets used and analysed during the current study can be obtained from the corresponding author on reasonable request.
